# Marine Protected Area Expansion and Country-Level Age-Standardized Adult Mortality

**DOI:** 10.1007/s10393-023-01658-3

**Published:** 2023-12-19

**Authors:** Sabrina S. Haque, Baylin J. Bennett, Thomas D. Brewer, Karyn Morrissey, Lora E. Fleming, Matthew O. Gribble

**Affiliations:** 1 Department of Environmental Health, Emory University Rollins School of Public Health, 1518 Clifton Road NE, Mailstop 1518-002-2BB, Atlanta, GA 30322 USA; 2 Department of Epidemiology, School of Public Health, University of Alabama at Birmingham, 1665 University Blvd, Birmingham, AL 35233 USA; 3 Australian National Centre for Ocean Resources and Security, Building 233, Innovation Campus, University of Wollongong, Wollongong, NSW 2522 Australia; 4 Division of Climate and Energy Policy, Department of Technology, Management and Economics, Technical University of Denmark, Anker Engelunds Vej 1 Bygning 101A, 2800 Kgs. Lyngby, Denmark; 5 European Centre for the Environment and Human Health, University of Exeter Medical School, Truro Cornwall, TR1 3HD UK; 6 Department of Medicine, Division of Occupational, Environmental and Climate Medicine, University of California, San Francisco, 490 Illinois Street, San Francisco, CA 94143 USA

**Keywords:** Environmental management, Environmental health, Ecosystem services, Fisheries, Conservation

## Abstract

Many countries have adopted targets to increase marine protected areas (MPAs) to limit the degradation of water bodies. Although there is evidence that MPAs can conserve marine life and promote biodiversity, there are limited data on the human health implications of MPAs. Using panel data from 1990, 2000, and 2014, we estimated the country-level associations between MPAs (i.e., percentage of territorial waters designated as marine reserves) and age-standardized mortality (i.e., age-standardized probability of dying between 15 and 60 years from all-causes among ages 15–60/100,000 population) by sex, among 110 countries. We fit mixed-effects linear regression models of mortality as a function of current MPA coverage, gross domestic product growth, year, the prior extent of MPA, electricity coverage, governance, and country-level random effects. We observed a significant inverse association between current MPA coverage and adult mortality. For each 5-percentage-point increase in current MPA coverage, a country had 0.982 times the geometric means of female and male mortality [geometric mean ratio: 0.982 (95% CI 0·976, 0·988)] conditional on past %MPA coverage and other modeled variables. The model showed no significant residual association of mortality with past %MPA conditional on current %MPA and other modeled variables. This is one of the first studies to show a positive association between increasing marine conservation and human health. This macro-level study suggests there may be important co-benefits for human health from expanding MPAs that merit further investigation.

**Supplementary Information:**

The online version contains supplementary material available at 10.1007/s10393-023-01658-3.

## Introduction

Ecosystem function and biological diversity are rapidly declining globally due to increasing human activity and climate change (National Academies of Sciences, Engineering, and Medicine, 2022). The United Nations (UN) estimates that 75% of land surface and 66% of oceans have been significantly altered, while 85% of wetlands have been lost (Díaz et al., 2019). These losses will substantially limit the availability of ecosystem services and goods, which are the natural ecosystem’s complex processes and tangible products that support human life and wellbeing (Daily, 1997).

Policymakers increasingly recognize the need to integrate environmental conservation into poverty alleviation, economic growth, and health improvement policies. Safeguarding marine environments and utilizing its resources sustainably have been argued to support several development goals including achieving food security, fostering livelihoods, and boosting climate resilience (Diz et al., 2019; Schleicher et al., 2018). Both national- and intergovernmental-level frameworks to achieve this integration have been put forward (Intergovernmental Science-Policy Platform on Biodiversity and Ecosystem Services, 2019; Pearson et al., 2019).

Spatially explicit land and water resource protection are commonly promoted environmental conservation interventions in global agendas, including the SDGs (United Nations, 2015). However, altering ecosystems (e.g., expanding agriculture, deforestation, building dams, etc.) are also motivated by economic and human development benefits to human populations. Therefore, there is a need to understand if environmental conservation interventions can complement, rather than conflict with, human development objectives to achieve better health and well-being outcomes.

Marine spatial planning (MSP; e.g., managing multiple uses of marine space) has proliferated in recent years due to awareness of accelerating marine degradation and relative political and legal expediency in implementing zoning policies (Douvere, 2008; Boonzaier and Pauly, 2016). A form of MSP, marine protected areas (MPAs) are geographically defined areas that are regulated and managed to achieve specific conservation objectives. MPAs originated in the 1970s in the Great Barrier Reef Marine Park in Australia as ‘no-take zones’ to protect the reef and help restore fish stocks, which are vital to marine communities that rely on fish for dietary and nutritional needs (Thilsted et al., 2016; Nölle, 2020). (Schaefer and Barale, 2011). Though implementation varies across regions, nearly 15% of marine areas under national jurisdiction are protected by MPA designation (Convention on Biological Diversity, 2017). Some MPAs are ‘no-take zones,’ while others regulate multiple uses of their resources. Evidence suggests that well-targeted, enforced, and long-term established MPAs have helped conserve marine life and promote biodiversity (Chape et al., 2005; Edgar et al., 2014). Several countries have adopted targets to increase MPAs as a proportion of their territorial waters to limit the degradation of specific water bodies that are valued. SDG Target 14.5 aims to achieve conservation of 10% of coastal and marine areas by 2020 globally (United Nations, 2015, 2022).

Nevertheless, MPAs as an effective conservation intervention have been met with criticism. A number of MPAs are not policed or well-enforced (so called “paper MPAs”); and many are established in locations that do not experience significant human disturbance (Santo, 2013; Pieraccini et al., 2017). MPAs can also displace, rather than prevent, environmental degradation to other unprotected areas (Sen, 2010).

One of the most severe criticisms of MPAs concerns the unintended consequences on local human communities. MPAs can inequitably impact populations that depend on protected areas for their livelihoods, food security, and nutrition, which may, in turn, deepen health and economic inequities (Cinner et al., 2012, 2014). For spatially explicit marine protection to endure as a management strategy, there is a need to identify how protected areas benefit human populations and whether the benefits outweigh the local and national costs.

A growing body of literature focuses on the relationship between marine biodiversity and ecosystem function with human health and well-being (World Health Organization and Secretariat of the Convention on Biological Diversity 2015; Bayles et al., 2016; Terraube et al., 2017; Ban et al., 2019; Fleming et al., 2019; Rasheed, 2020). For instance, the loss of coastal barrier systems (e.g., coastal mangroves, coral reefs, wetlands, and vegetated dunes) is linked to strengthened storm surges and increased morbidity and mortality in coastal communities from storms and sea-level rise (Dahdouh-Guebas et al., 2005; Kunkel et al., 2006; Myers et al., 2013). Coastal systems may affect the environmental distribution of pollutants and pathogens, which may be especially important for the health of populations that are in contact with surface water during recreational or domestic activities (Brauman et al., 2007; Pattanayak and Wendland, 2007; Myers et al., 2013). The loss of marine ecosystems can affect social well-being and mental health, particularly of local communities whose culture is intrinsically tied to marine spaces (White et al., 2010, 2016; Martin et al., 2016).

Deteriorating marine systems also have an impact on food security and livelihoods. The demand for seafood is projected to rise over the coming decades, and sustainable management approaches to prevent fisheries collapse can help increase net supply of foods (Costello et al., 2020). Fish alone accounts for more than 15% of animal protein consumption globally and even more for coastal communities (Food and Agriculture Organization, 2018), although access to fish varies geographically (Beveridge et al., 2013). Consumption of fish high in micronutrients and low in contaminants has been associated with potential cardiovascular, neurological, and other health benefits (Gribble et al., 2016); nutrients from fish can help combat micronutrient deficiencies in some settings (Nölle et al., 2020). Further, about 200 million jobs are directly or indirectly related to the fishing sector, with a large proportion of these jobs in the poorest and least developed countries (Food and Agriculture Organization, 2018); fish industry contributions to income are thereby indirectly important for food security for more than 10% of the world’s population particularly in lower-income countries (Béné et al., 2015).

A systematic review of the effects of MPAs on fisheries found that food security stayed relatively stable or increased in smaller and older MPAs, and generally increased the political power of fishing communities (Mascia et al., 2010). A more recent review of 118 peer-reviewed analyses on the effects of MPAs also found more positive than negative well-being outcomes in the literature (Ban et al., 2019). A variety of positive well-being outcomes were observed, including but not limited to income, food security, mental health, social capital, and community empowerment; however, positive outcomes may co-occur with negative well-being outcomes (e.g., increased conflict and management costs) (Ban et al., 2019).

Although there is increasing appreciation for the societal benefits of MPAs, systematic reviews uniformly identify the need for additional research on the health and well-being implications of environmental conservation interventions to inform more just and sustainable development policy (Martin et al., 2016; Ban et al., 2019; Rasheed, 2020). While some localized assessments have shown limited evidence of positive effects of MPAs on nutritional status (Gjertsen, 2005; Aswani and Furusawa, 2007), and various studies have shown social, economic, and cultural benefits (Martin et al., 2016; Ban et al., 2019; Rasheed, 2020), this is the first study to longitudinally examine, at a global scale, the association of marine protection with population health.

The objective of this study was to characterize the relationship between MPAs and age-standardized adult sex-specific mortality (i.e., age-standardized probability of dying from all causes among ages 15–60 per 1000 population) across countries using publicly available panel data spanning a 34-year window. Age-standardized adult mortality was chosen as a health metric because it is relatively reliable at a national scale and potentially aggregates the distinct benefits of marine conservation over the life course (e.g., strengthening food and water security, reducing infectious disease, improving mental health, and increasing resilience to climate change and natural disasters).

## Materials and Methods

### Data Sources

This is a secondary data analysis of four publicly available datasets. Country-level data on marine protected areas as a percentage of territorial waters were downloaded from the UN Statistics Division which hosted data for the years 1990, 2000, and 2014 (United Nations, 2016). Percentage of population living within 100 km (km) from coastlines were downloaded from the Socioeconomic Data and Applications Center (SEDAC) of Columbia University. All other variables were subsequently obtained for these same years. Country-level adult female and male mortality (i.e., age-standardized probability of dying between the ages of 15 and 60 from all-causes among per 1000 population), GDP growth and electricity access were obtained from the World Bank Development Indicators Database (The World Bank, 2022). The World Bank obtained age-standardized mortality from the UN Population Division, UC Berkley, and Max Planck Institute for Demographic Research. “Voice and accountability” estimates were obtained from the Governance Indicator dataset authored by the World Bank (The World Bank, 2018).

These data were merged into one dataset and analyzed using Stata/MP 17.0. Only countries with > 50% of the population living within 100 km of the coastline were included in the analysis, giving a sample of 134 countries. This exclusion was made since the underlying hypothesis for observing effects on adult mortality is based on spillover benefits of MPAs to communities living near coastal areas. Of the 134 countries, 110 had appropriate national-level mortality data available. All variables, including the mortality outcomes, were modeled as continuous.

### Statistical Approach

We fit mixed-effects linear regression models of log-transformed mortality as a function of the percentage of territorial waters reserved as MPAs with normal random effects by country intercepts and cluster-robust standard errors (Sribney, 2022). Models were fit separately for overall mortality and sex-specific mortality. First, we fit a model for current extent of MPA coverage with no regression adjustment (Model 1). We then adjusted for the extent of MPA coverage a decade prior (Model 2), then further adjusted for year (Model 3), further adjusted for GDP growth and proportion of the country with electricity access (Model 4), and finally also adjusted for voice and accountability (Model 5). GDP growth and electricity coverage are measure for economic growth, and stages of development experienced that may affect mortality (Tresserras et al., 1992; Backlund et al., 1996; Wang, 2003). Voice and accountability measures distinguish between countries in governance that may affect health and well-being, specifically measuring “the extent to which a country’s citizens are able to participate in selecting their government, and to enjoy freedom of expression, freedom of association, and a free media” (page 4, (Kaufmann et al., 2011)).

Multiple imputation was performed for missing data on GDP growth (*n* = 95 country-year observations imputed) using linear regression, and for electricity coverage (*n* = 64 country-year observations imputed) and voice and accountability estimate (*n* = 184 country-year observations imputed) using predictive mean matching, conditional on country, GDP growth, electricity coverage, marine protected area coverage (current and lagged), year, female mortality, and male mortality, using chained equations, generating 200 imputed datasets (Little, 1988; Azur et al., 2011).

Although marine protected area coverage and male and female mortality are in the multiple imputation model to generate plausible values of the adjustment variables in imputed datasets, our final analysis was restricted to records with complete data on MPA coverage and mortality, with imputed values of the adjustment variables.

The model forms for our main data analysis are as follows:

$$ \begin{aligned} & {\text{Model}}\,{1:}\,Log\left( {Age - standardized\,mortality} \right)_{{{\varvec{country}}\,\varvec{j,observation}\,\varvec{i,time}\,{\varvec{t}}}} =\upbeta _{o} +\upbeta _{1} \left( {\% MPA} \right)_{{\varvec{j,i,t}}} +\upxi _{j} +\upvarepsilon _{j,i} \\ & {\text{Model}}\,{2:}\,Log\left( {Age - standardized\,mortality} \right)_{{{\varvec{country}}\,\varvec{j, observation}\,\varvec{i, time}\,{\varvec{t}}}} =\upbeta _{o} +\upbeta _{1} \left( {\% MPA} \right)_{{\varvec{j,i,t}}} \\ & \quad \quad +\upbeta _{2} \left( {\% MPA} \right)_{{\varvec{j,i,t} - 1}} +\upxi _{j} +\upvarepsilon_{j,i} \\ & {\text{Model}}\,{3:}\,Log\left( {Age - standardized\,mortality} \right)_{{{\varvec{country}}\,\varvec{j,observation}\,\varvec{i,time}\,{\varvec{t}}}} =\upbeta _{o} +\upbeta _{1} \,\left( {\% MPA} \right)_{{\varvec{j,i,t}}} \\ & \quad \quad +\upbeta _{2} \left( {\% MPA} \right)_{{\varvec{j,i,t} - 1}} +\upbeta _{3} \left( {year} \right)_{{\varvec{j,i,t}}} + \xi_{j} +\upvarepsilon_{j,i} \\ & {\text{Model}}\,{4:}\,Log\left( {Age - standardized\,mortality} \right)_{{{\varvec{country}}\,\varvec{j,observation}\,\varvec{i,time}\,{\varvec{t}}}} =\upbeta _{o} +\upbeta _{1} \left( {\% MPA} \right)_{{\varvec{j,i,t}}} \\ & \quad \quad +\upbeta _{2} \left( {\% MPA} \right)_{{\varvec{j,i,t} - 1}} +\upbeta _{3} \left( {year} \right)_{{\varvec{j,i,t}}} +\upbeta _{4} \left( {\% GDPgrowth} \right)_{{\varvec{j,i,t}}} +\upbeta _{5} \left( {electricity\,coverage} \right)_{{\varvec{j,i,t}}} +\upxi _{j} +\upvarepsilon_{j,i} \\ & {\text{Model}}\,{5:}\,Log\left( {Age - standardized\,mortality} \right)_{{{\varvec{country}}\,\varvec{j,observation}\,\varvec{i,time}\,{\varvec{t}}}} =\upbeta _{o} +\upbeta _{1} \left( {\% MPA} \right)_{{\varvec{j,i,t}}} \\ & \quad \quad +\upbeta _{2} \left( {\% MPA} \right)_{{\varvec{j,i,t - }1}} +\upbeta _{3} \left( {year} \right)_{{\varvec{j,i,t}}} +\upbeta _{4} \left( {\% GDPgrowth} \right)_{j,i,t} +\upbeta _{5} \left( {electricity\,coverage} \right)_{j,i,t} \\ & \quad \quad +\upbeta _{6} \left( {voice\,and\,accountability\,estimate} \right)_{{\varvec{j,i,t}}} +\upxi _{j} +\upvarepsilon _{j,i} \\ &\upxi \sim N\left( {0,\uptau ^{2} } \right) \\ &\upvarepsilon \sim N\left( {0,\upsigma ^{2} } \right) \\ \end{aligned} $$

where *j* is the country for observation *i*, observed at time *t* (or *t − *1), *β*_*o*_ is the grand mean of log mortality, *ξ*_*j*_ is the normally-distributed random intercept for country *j* (variance *τ*^2^), *ε*_*j,i*_ is the deviation from the local expected value (assumed normal with variance *σ*^2^*)*, *β*_1,_ *β*_2,_ *β*_3,_ *β*_4_, *β*_5,_ and *β*_6_ are fixed effect regression coefficients for covariates evaluated at *j*,*i*,*t*.

In secondary analyses (Models 6, 7, 8), we reparametrized some of the predictor variables as change scores (e.g., *∆*%MPA_t_ = %MPA_t_ − %MPA_t-1_).

$$ \begin{aligned} & {\text{Model}}\,{6:}\,Log\left( {Age - standardized mortality} \right)_{{{\varvec{country}}\,\varvec{j,observation}\,\varvec{i,year}\,{\varvec{t}}}} =\upbeta _{o} +\upbeta _{1} (\Delta \% MPA)_{{\varvec{j,i,t}}} +\upbeta _{2} \left( {GDPgrowth} \right)_{{\varvec{j,i,t}}} \\ & \quad \quad +\upbeta _{3} \left( {{\text{electricity}}\,{\text{coverage}}} \right)_{{\varvec{j,i,t}}} +\upbeta _{4} \left( {voice\,and\,accountability\,estimate} \right)_{{\varvec{j,i,}t}} +\upxi _{j} +\upvarepsilon _{j,i} \\ & {\text{Model}}\,{7:}\,Log\left( {Age - standardized mortality} \right)_{{{\varvec{country}}\,\varvec{j,observation}\,\varvec{i,year}\,{\varvec{t}}}} =\upbeta _{o} +\upbeta _{1} \left( {\Delta \% MPA} \right)_{{\varvec{j,i,t}}} +\upbeta _{2} \left( {year} \right)_{{\varvec{j,i,t}}} \\ & \quad \quad +\upbeta _{3} \left( {GDPgrowth} \right)_{{\varvec{j,i,t}}} +\upbeta _{4} \left( {electricity\,coverage} \right)_{{\varvec{j,i,t}}} +\upbeta _{5} \left( {voice\,and\,accountability\,estimate} \right)_{{\varvec{j,i,t}}} +\upxi _{j} +\upvarepsilon _{j,i} \\ & {\text{Model}}\,{8:}\,Log\left( {Age - standardized mortality} \right)_{{{\varvec{country}}\,\varvec{j,observation}\,\varvec{i,year}\,{\varvec{t}}}} =\upbeta _{o} +\upbeta _{1} \left( {\Delta \% MPA} \right)_{{\varvec{j,i,t}}} +\upbeta _{2} \left( {\Delta GDPgrowth} \right)_{{\varvec{j,i,t}}} \\ & \quad \quad +\upbeta _{3} \left( {\Delta electricity\,coverage} \right)_{{\varvec{j,i,t}}} +\upbeta _{4} \left( {\Delta voice\,and\,accountability\,estimate} \right)_{{\varvec{j,i,t}}} +\upxi _{j} +\upvarepsilon _{j,i} \\ \end{aligned} $$

In a final sensitivity analysis, we implemented a ‘first difference’ estimator to assess the relationship of change in mortality to change in all other predictors. This model did not include random effects, but did include robust standard errors. This model also included change in year.

$$ \begin{aligned} & {\text{Model}}\,{9:}\,Log\left( {Age - standardized mortality} \right)_{{{\varvec{country}}\,\varvec{j,observation}\,\varvec{i,year}\,{\varvec{t}}}} - Log\left( {Age - standardized mortality} \right)_{{{\varvec{country}}\,\varvec{j,observation}\,\varvec{i,year}\,{\varvec{t}}{ - 1}}} \\ & \quad =\upbeta _{o} +\upbeta _{1} \left( {\Delta \% MPA} \right)_{{\varvec{j,i,t}}} +\upbeta _{2} \left( {\Delta \% year} \right)_{{\varvec{j,i,t}}} +\upbeta _{3} \left( {\Delta GDPgrowth} \right)_{{\varvec{j,i,t}}} +\upbeta _{4} \left( {\Delta electricity\,coverage} \right)_{{\varvec{j,i,t}}} \\ & \quad \quad +\upbeta _{5} \left( {\Delta voice\,and\,accountability\,estimate} \right)_{{\varvec{j,i,t}}} +\upvarepsilon _{j,i} \\ \end{aligned} $$

All analysis was carried out using Stata/MP 17.0 and the analysis code is available in the supplementary material.

## Results and Discussion

A total of 134 countries had data on MPA coverage for the years 1990, 2000, or 2014, and had at least 50% of the population living within 100 km of the coastline. Among these countries, 110 had accessible national sex-segregated adult mortality data.

**Figure 1:**
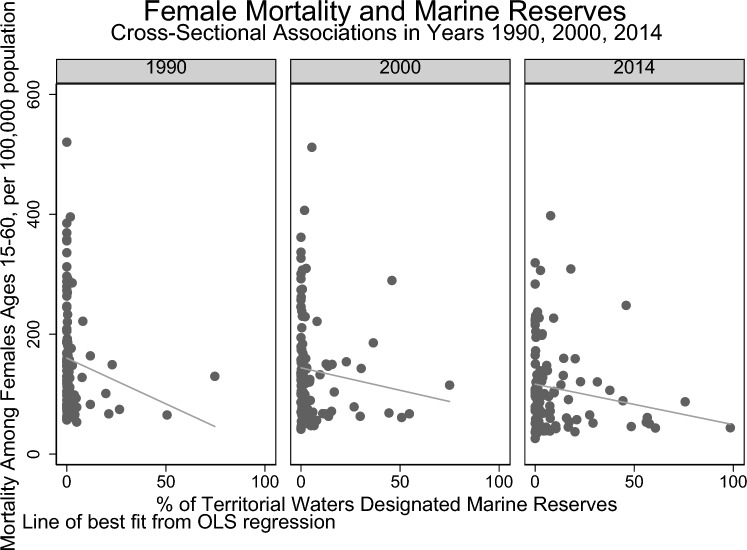
Cross-sectional unadjusted associations of standardized mortality (among females, aged 15–60, per 100,000 population) and Marine Reserves (% of territorial waters as marine protected areas *n* = 322).

**Figure 2:**
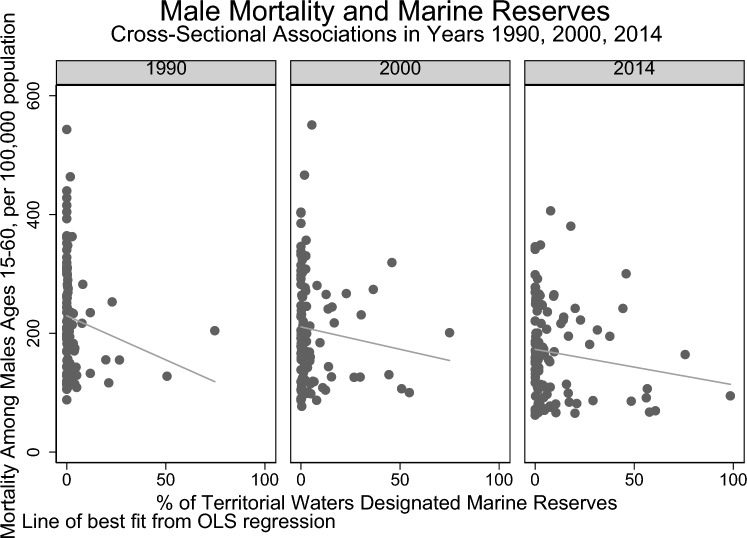
Cross-sectional unadjusted associations of standardized mortality (among males, aged 15–60, per 100,000 population) and Marine Reserves (% of territorial waters as marine protected areas *n* = 322).

Among the countries included in our database, there was a general decrease in age-standardized mortality for both sexes across the years 1990, 2000, and 2014 (Table 1). At the same time, there were also concurrent increases in the extent of MPAs. In 1990, the mean extent of MPAs (percentage [%] of territorial waters) was 2.89% (SE 9.18%); in 2000, 5·43% (SE 11.83%); and in 2014, 10·09% (SE 18.25%). Thus, descriptive statistics indicate that at a global level, as marine protection increases, the age-standardized mortality is also declining across countries over 1990–2014. There is an inverse relationship between the extent of marine protection coverage and overall age-standardized mortality across countries within each year (Figs. 1 and 2).

**Table 1:** Summary of Indicators.

Year	1990	2000	2014
Female mortality (mean deaths per 1000 people, SD)	155.49, ± 89.39 *N* = 108	139.37, ± 88.12 *N* = 110	109.59, ± 73.79 *N* = 104
Male mortality (mean deaths per 1000 people, SD)	224.99, ± 91.34 *N* = 108	206.12, ± 91.57 *N* = 110	166.70, ± 81.49 *N* = 104
Marine protected area (mean % of territorial waters, SD)	2.89, ± 9.18 *N* = 134	5.43, ± 11.83 *N* = 134	10.09, ± 18.25 *N* = 134
GDP growth (mean, SD)	2.65, ± 7.56 *N* = 93	4.60, ± 4.92 *N* = 108	2.97, ± 4.10 *N* = 106
Electricity coverage (mean, SD)	77.15, ± 29.25 *N* = 104	81.72, ± 27.04 *N* = 114	86.26, ± 25.65 *N* = 120
Voice and accountability estimate	–	0.29, ± 0.94 *N* = 108	0.24 ± 0.97 *N* = 110

Using mixed-effects linear regression models, Table 2 presents the results of pooled bivariate cross-sectional analysis of 110 countries (*n* = 322 observations across years), considering only contemporary marine protection (e.g., univariate comparisons) and the age-standardized mortality. Here, we observed an inverse association between the extent of marine protected areas (% of territorial waters) and the age-standardized mortality among persons between the ages of 15 and 60 per 100,000 population. The geometric mean ratio (GMR) for female age-standardized adult mortality was 0·952 (95% confidence interval (CI) 0·936, 0·968); for males 0·955 (95% CI 0·943, 0·967) (Model 1, Table 2). The MPA variable is measured in 5-percentage-point increases. This means that for every 5-percentage-point increase in MPAs, a country has approximately 0·952 times the geometric mean ratio of mortality for females and 0·955 times for males (Table 2).

**Table 2:** Multivariable Associations of Contemporary Marine Protection (% of Territorial Waters) and Mortality (*N* = 110 Countries).

Model	Female mortality (GMR, 95%)	Male mortality (GMR, 95%)
Model 1 *n* = 322^a^	0.952 (0.936, 0.968)	0.955 (0.943, 0.967)
Model 2 *n* = 214^b^	0.963 (0.951, 0.976)	0.967 (0.958, 0.975)
Model 3 *n* = 214^c^	0.985 (0.979, 0.991)	0.983 (0.977, 0.989)
Model 4 *n* = 214^d^	0.981 (0.975, 0.988)	0.982 (0.975, 0.988)
Model 5 *n* = 214^e^	0.982 (0.976, 0.988)	0.982 (0.976, 0.988)

^a^Model 1 is a simple model that only conditions contemporary MPA and mortality on country-level random effects and cluster-robust standard errors.

^b^Model 2 further adjusts for legacy of MPA (past % MPA of territorial waters).

^c^Model 3 further adjusts for calendar year.

^d^Model 4 further adjusts for development indicators including contemporaneous % GDP growth and electricity coverage.

^e^Model 5 further adjusts for governance indicator including a voice and accountability estimate.

After considering both past and contemporary marine protection in a jointly adjusted model (Model 2, Table 2), there was no significant association of past percentage marine protected areas with contemporary mortality, conditional on the current year, %GDP growth, electricity coverage, governance (e.g., voice and accountability estimates), and country-level random effects (*N* = 110 countries and *n* = 214 observations; this analysis required at least two time-points per country).

However, despite the lack of association between contemporary mortality with the legacy of past protection observed in Model 2, Model 5 finds a significant relationship between contemporary mortality and contemporary %MPA, for both females (GMR 0·982: 95% CI 0·976, 0·988) and males (0.982: 95% CI 0·976, 0·988), conditional on past %MPA, the current year, %GDP growth, electricity coverage, voice accountability estimates, and country-level random (Table 2). In other words, for every 5-percentage-point increase in MPAs, a country has approximately 0·984 times the geometric mean ratio of mortality for females and 0.983 times for males (Table 2). These results indicate that current MPAs have an inverse association with both female and male mortality adjusted for time, country-level economic, governance characteristics, and random effects with standard errors.

The interpretation of current %MPA conditional on past %MPA is the change over time in %MPA. Therefore, to reduce the number of parameters in the model and improve estimation precision, predictor variables were recoded as “change scores” on 5 percentage point scale (e.g., current %MPA − past MPA%) as secondary analyses (Supplemental Table 1). There were similar results, with significant associations between contemporary age-standardized mortality and MPA expansion (i.e., %MPA change score). For example, in the model conditional on the current year, %GDP growth, electricity coverage, governance (voice accountability estimates), and country-level random effects, among females, the GMR for %MPA change score was 0·986 (95% CI 0·979, 0·992) and among males, 0·986 (95% CI 0·978, 0·994) (Supplemental Table 1). In the first difference estimator models, the GMR of change score per %MPA change score for log-mortality for females was 0.990 (95% CI 0.981, 0.998) and for males was 0.987 (95% CI 0.980, 0.994), conditional on change in other modeled variables.

Higher marine conservation (as measured by the percentage of territorial waters defined as marine reserves) was positively associated with decreased age-standardized mortality for adults between the ages 15 and 60, from 1990 to 2014. This was seen even after adjustment for economic growth (as measured by % growth in the gross domestic product), year, electricity coverage, and governance (“voice” and accountability estimate). Although there was a crude inverse relationship between legacy MPA coverage and contemporary mortality, legacy MPA coverage did not have a significant association with mortality in the adjusted model. However, this could be variance inflation from adjustment of other variables. This could also result from measurement error in defining legacy MPA coverage. MPA data was only available at three-time points, 10–14 years apart. The contemporary MPA measurements could reflect MPA coverage in the latter part of the time period between visits and capture the rapid effects accruing in shorter time periods.

As a macro-level study, these findings are subject to the limitations of the study design; in particular, one needs to be cautious of drawing any inferences about an individual or local community risks from aggregated country-level mortality data (Piantadosi et al., 1988). This analysis looks at national population effects and cannot study effects on the most affected communities that live adjacent to marine spaces. As an observational study based on countries with available data, risk of bias from unmeasured confounding and selection bias are possible.

Our analysis recoding the MPA exposure variable as a change score produced similar results. However, we recognize that the covariates are not similarly time-lagged and have linear continuous parameterization of continuous covariates, which allows residual confounding from model misspecification. Nonetheless, the association between increased MPA coverage and reduced mortality is interesting and may suggest important public health benefits from marine conservation policies that merit further investigation.

A possible explanation consistent with our findings is that the presence of an MPA has a beneficial influence on human health, perhaps from livelihood, tourism economic benefits, improved mental well-being, decreased infectious disease incidence, and/or increased climate resilience (Keesing et al., 2010; Myers et al., 2013; Santo, 2013; Biggs et al., 2016).

Limited external support for the MPAs’ association with nutrition comes from a cross-sectional case study in the Solomon Islands, where villages with MPAs had better nutrition (e.g., higher protein and energy intake) than villages without marine protected areas (Aswani and Furusawa, 2007). However, in a matched analysis of coral reef fishing communities in Kenya, the presence of marine reserves was not associated with food security (Darling, 2014), as measured by protein consumption, dietary diversity, or an index of food coping strategies. The Kenyan case study did not show evidence that fishing households near reserves had different nutrition than fishing households far from reserves, but this test for interaction was not statistically well-powered given the limited number of fishing households in the study (*n* = 58). We hope more detailed, local-level longitudinal case data on MPA qualities and various health and wellbeing outcomes may be available in the future to provide better context for these contradictory local stories and provide a clearer picture of on-the-ground relationships.

There may also be plausible mental health benefits from MPAs. A recent systematic review found many studies associate “green space” with lower cardiovascular disease mortality, but found no studies yet on “blue spaces” (Gascon et al., 2016). However, there is growing interest in, and evidence for, the potential psychological benefits of “blue space” (White et al., 2010, 2016, 2017; Völker and Kistemann, 2011; Cracknell et al., 2016, 2017) which increasingly appear to be tied to perceived environmental quality.

A survey of beachgoers in California found that psychological perceptions (e.g., perceived temperature) were much stronger predictors of positive mental health scores than were empirical conditions (e.g., measured temperature) (Hipp and Ogunseitan, 2011); and a photograph-exposure intervention study found that images of trash appeared to reduce the positive mental health benefits from coastal scenes (Wyles et al., 2015). There is also limited psychometric research suggesting positive psychological benefits from volunteer participation in wildlife monitoring programs in marine protected areas (Koss and Kingsley, 2010). Therefore, we speculate that the perception of an improved environmental quality from the expansion of marine protected areas might amplify “blue space” psychological benefits, and thereby potentially reduce allostatic load and possibly, cardiovascular disease mortality. This posited mechanism is largely speculation at this stage but could be interrogated through future research.

An additional explanation to the association of marine protected areas and mortality is that improved marine protection is a marker for a changing social context (e.g., improving economy or changing values) in the country, which may have other pathways leading to reduced mortality especially among females than an effect directly mediated by exposure to increased protected marine areas. However, adjusting our models for growth in %GDP, electricity coverage, and governance, did not substantially affect our associations.

The results are limited by its observational study design and sample size, making the relationship between MPAs and mortality vulnerable to residual confounding. Although the adjusted models did control for a set of development, socio-economic, and governance variables that simultaneously relate to MPA policies and public health, the model cannot provide results that would imply causal effects of increased MPAs. We note an overall decrease in mortality across our studied time period, as well as an increase over this time period in the extent of marine protected areas, so there may be residual confounding by temporal trends in a causal variable that we did not adjust for even after adjusting for calendar year, contemporaneous % growth in GDP, electricity coverage, and governance.

We would like to highlight the limitations of our results given the study’s observational study design and small sample size and emphasize the importance and value of future additional data collection on marine conservation and human well-being. We were constrained in our analysis to only three waves of historical data (effectively two waves for analysis when modeling lagged exposures) in the compiled international assessments of marine protection and mortality outcomes in 1990, 2000, and 2014. These multinational snapshots are not the only years that specific countries kept records but are the only years available in the dataset we used, and limitations on country-years available for analysis induce sample size constraints that make it difficult for statistical models to truly account for time-varying confounding or allow robust examination of potentially nonlinear and heterogeneous relationships.

In addition to encouraging completion of a larger retrospective multinational dataset that could include more years through greater knowledge-sharing (Poto et al., 2022), our analysis is also limited to the coverage of MPAs and does not consider quality dimensions (e.g., age, enforcement, isolation) that have been deemed important for the effectiveness on conservation outcomes (Edgar et al., 2014); the size of a protected area (be it in absolute terms like square kilometers, or relative terms such as % of territorial waters) is not the only axis for measuring quality of an MPA (Chape et al., 2005); and the MPA framework has been criticized as not consistent across all sites in attaining conservation goals (Jameson et al., 2002; Agardy et al., 2003; Sale et al., 2005). There is a growing body of work on predicting success for conservation goals (Fox et al., 2012; Edgar et al., 2014), including the importance of an appropriate governance structure for each MPA (McCay and Jones, 2011); and there is notable variation in the quality of habitat to be protected such that replacing under-performing protected areas with better areas of the same size can improve conservation outcomes (Fuller et al., 2010). Additional detail on the social, political, and biological characteristics of specific MPAs might improve future analyses trying to understand mechanisms of why the expansion of marine protected areas is associated with reduced mortality.

As interest grows toward the “30 by 30 Initiative” (the goal to cover at least 30% of the global ocean in MPAs by 2030 (O’Leary et al., 2016)), we think that additional prospective data collection during the rapid further expansion of MPAs could be possible and invaluable for future evaluations. This work highlights some of the potential confounding factors that might be more thoroughly and prospectively assessed if data are collected consistently and comprehensively in real-time. Further, we think that subnational (e.g., regional) data, and supranational data (e.g., the anticipated emergence of multinational marine protected areas), on specific health outcomes as well as the all-cause mortality under current study, could allow for greater insights than are possible from three (temporally distant) years’ data on country-level vital statistics. Further, there would be significant value in exploring associations using person-level (e.g., cohort) data rather than spatially aggregated vital statistics data.

Another important issue is the availability and definitions of health measures. For example, a systematic review found limited empirical analyses quantifying the relationship between MPAs and mortality, and identified variations in defining well-being (Rasheed, 2020). Future MPA studies should attempt to follow consistent frameworks of well-being and use standardized indicators to promote generalizability and cross-comparison (Rasheed, 2020).

## Conclusion

This macro-level study found an inverse association between expanding MPAs and human mortality across multiple countries that merits further investigation. We encourage public health and marine policymakers to regard this preliminary evidence with some skepticism, but also to contemplate the possibility that there could be benefits beyond marine species conservation from protective fisheries policies. We also encourage policymakers and managers to track indices of local community health and well-being as part of their local program evaluations.

### Supplementary Information

Below is the link to the electronic supplementary material.

Supplementary file1 (DOCX 37 kb)
