# Correction: Marine Protected Area Expansion and Country-Level Age-Standardized Adult Mortality

**DOI:** 10.1007/s10393-023-01672-5

**Published:** 2024-01-17

**Authors:** Sabrina S. Haque, Baylin J. Bennett, Thomas D. Brewer, Karyn Morrissey, Lora E. Fleming, Matthew O. Gribble

**Affiliations:** 1 Department of Environmental Health, Emory University Rollins School of Public Health, 1518 Clifton Road NE, Mailstop 1518-002-2BB, Atlanta, GA 30322 USA; 2 Department of Epidemiology, School of Public Health, University of Alabama at Birmingham, 1665 University Blvd, Birmingham, AL 35233 USA; 3 Australian National Centre for Ocean Resources and Security, Building 233, Innovation Campus, University of Wollongong, Wollongong, NSW 2522 Australia; 4 Division of Climate and Energy Policy, Department of Technology, Management and Economics, Technical University of Denmark, Anker Engelunds Vej 1 Bygning 101A, 2800 Kgs. Lyngby, Denmark; 5 European Centre for the Environment and Human Health, University of Exeter Medical School, Truro Cornwall, TR1 3HD UK; 6 Department of Medicine, Division of Occupational, Environmental and Climate Medicine, University of California, San Francisco, 490 Illinois Street, San Francisco, CA 94143 USA

**Correction to: EcoHealth** 10.1007/s10393-023-01658-3

In this article the affiliation details for Baylin J. Bennett, Thomas D. Brewer and Lora E. Flemming were incorrectly given as 'European Centre for the Environment and Human Health, University of Exeter Medical School, Truro Cornwall TR1 3HD, UK, Department of Epidemiology, School of Public Health, University of Alabama at Birmingham, 1665 University Blvd, Birmingham, AL 35233 and Australian National Centre for Ocean Resources and Security, Building 233, Innovation Campus, University of Wollongong, Wollongong, NSW 2522, Australia' but should have been 'Baylin J. Bennett (Department of Epidemiology, School of Public Health, University of Alabama at Birmingham, 1665 University Blvd, Birmingham, AL 35233), Thomas D. Brewer (Australian National Centre for Ocean Resources and Security, Building 233, Innovation Campus, University of Wollongong, Wollongong, NSW 2522, Australia) and Lora E. Flemming (European Centre for the Environment and Human Health, University of Exeter Medical School, Truro Cornwall TR1 3HD, UK). The original article has been corrected.

