# Supplementary material for: Marine Protected Area Expansion and Country-Level Age-Standardized Adult Mortality
Source: Ecohealth. 2023 Dec 19;20(3):236–48. doi: 10.1007/s10393-023-01658-3 (PMC10757699; doi:10.1007/s10393-023-01658-3)
Supplement: Supplementary file 1 — Supplementary file1 (DOCX 37 kb) [file 10393_2023_1658_MOESM1_ESM.docx]

**Supplemental Table 1. Multivariable associations of increase in relative marine protection from the prior period to contemporary period (~ one decade) in regressions conditional on country-level random effects, and with cluster-robust standard errors (N=105, n=115). Due to lagged predictors, these analyses considered sex-specific mortality outcomes in 2000 and 2014, and predictor data from 1990, 2000, or 2014. There were 200 imputed datasets used for the analysis.**

| **Model** | **Exposure** | **Adjustment Variables** | **Female Mortality  (GMR, 95%) RVI, FMI** | **Male Mortality  (GMR, 95%) RVI, FMI** |
| --- | --- | --- | --- | --- |
| Model 6 n= 115 | Δ %MPA from last period to current year | contemporary %GDP growth, electricity coverage, voice and accountability estimates | 0.978 (0.965, 0.991) | 0.980 (0.967, 0.993) |
| Model 7 n= 115 | Δ %MPA from last period to current year | contemporary %GDP growth, electricity coverage, voice and accountability estimates, current year | 0.986 (0.979, 0.992) | 0.986 (0.978, 0.994) |
| Model 8 n= 115 | Δ %MPA from last period to current year | Δ GDP growth, Δ electricity coverage, Δ voice and accountability estimates | 0.986 (0.951, 1.023) | 0.996 (0.964, 1.029) |

# **Supplementary Material**

***Stata Code***

*Load in the data

use "mpa_data.dta", clear

encode Country, gen(pais)

gen pais2 = pais

sort pais year

by pais: gen lag_dif_mpa = MAPTW-MAPTW[_n-1]

by pais: gen lag1_mpa = MAPTW[_n-1]

gen c_year = year-2000

egen countrynumber = group(Country)

list pais countrynumber in 1/10, sepby(pais)

**MPA by 5

gen MAPTW_5=MAPTW/5

**lag by 5

by pais: gen lag_dif_5_mpa = MAPTW_5-MAPTW_5[_n-1]

by pais: gen lag1_5_mpa = MAPTW_5[_n-1]

**log-tranformed mortality rates

gen logmortmale=log(mortmale)

gen logmortfemale=log(mortfemale)

* Make some placeholder values for the mortality and MPA variables, can use these for imputation model

* for other variables (e.g., MAR missingness not MCAR) but then analyze complete case on those variables

gen c_yearb = c_year

gen MAPTW_5b = MAPTW_5

gen lag1_5_mpab = lag1_5_mpa

gen logmortmaleb = logmortmale

gen logmortfemaleb = logmortfemale

**imputations

mi set mlong

mi register imputed vae GDPgrowth elc pais2 c_yearb MAPTW_5b lag1_5_mpab logmortmaleb logmortfemaleb

mi register regular c_year MAPTW_5 lag1_5_mpa logmortmale logmortfemale

mi impute chained (pmm, knn(15)) vae (regress) GDPgrowth (regress) c_yearb (regress) MAPTW_5b (regress) lag1_5_mpab (regress) logmortmaleb (regress) logmortfemaleb (pmm, knn(15)) elc (mlogit) pais2, add(200) rseed(123)

mi passive: by pais: gen lag_dif_5_GDPgrowth = GDPgrowth-GDPgrowth[_n-1]

mi passive: by pais: gen lag1_5_GDPgrowth = GDPgrowth[_n-1]

mi passive: by pais: gen lag_dif_5_elc = elc-elc[_n-1]

mi passive: by pais: gen lag1_5_elc = elc[_n-1]

mi passive: by pais: gen lag_dif_5_vae = vae-vae[_n-1]

mi passive: by pais: gen lag1_5_vae = vae[_n-1]

*lag mortality

mi passive: by pais: gen lag1_malemort = logmortmale[_n-1]

mi passive: by pais: gen lag1_femalemort = logmortfemale[_n-1]

mi passive: gen lag1_year = c_year[_n-1]

mi passive: gen clagyear = c_year - lag1_year

mi passive: gen lagdifmale = logmortmale - lag1_malemort

mi passive: gen lagdiffemale = logmortfemale - lag1_femalemort

*Now mi xtset the data so we can use xtreg

mi xtset pais

*Models

*Model 1 simple

mi estimate: xtreg logmortmale MAPTW_5 , re vce(robust)

mi estimate: xtreg logmortfemale MAPTW_5 , re vce(robust)

*Model 2 further adjusts for legacy of MPA

mi estimate: xtreg logmortmale MAPTW_5 lag1_5_mpa , re vce(robust)

mi estimate: xtreg logmortfemale MAPTW_5 lag1_5_mpa , re vce(robust)

*Model 3 further adjusts for calendar year

mi estimate: xtreg logmortmale MAPTW_5 lag1_5_mpa c_year , re vce(robust)

mi estimate: xtreg logmortfemale MAPTW_5 lag1_5_mpa c_year , re vce(robust)

*Model 4 further adjusts for development indicators including contemporaneous % GDP growth and electricity coverage

mi estimate: xtreg logmortmale MAPTW_5 lag1_5_mpa c_year GDPgrowth elc , re vce(robust)

mi estimate: xtreg logmortfemale MAPTW_5 lag1_5_mpa c_year GDPgrowth elc , re vce(robust)

*Model 5 further adjusts for governance indicator including a voice and accountability estimate

mi estimate: xtreg logmortmale MAPTW_5 lag1_5_mpa c_year GDPgrowth elc vae , re vce(robust)

mi estimate: xtreg logmortfemale MAPTW_5 lag1_5_mpa c_year GDPgrowth elc vae , re vce(robust)

*Sensitivity analysis: Differencing Model (only includes data from 2000 and 2014 not 1990 since no lagged exposure defined in 1990)

mi estimate: xtreg logmortfemale lag_dif_5_mpa GDPgrowth elc vae, re vce(robust)

mi estimate: xtreg logmortmale lag_dif_5_mpa GDPgrowth elc vae, re vce(robust)

mi estimate: xtreg logmortfemale lag_dif_5_mpa c_year GDPgrowth elc vae, re vce(robust)

mi estimate: xtreg logmortmale lag_dif_5_mpa c_year GDPgrowth elc vae, re vce(robust)

mi estimate: xtreg logmortfemale lag_dif_5_mpa lag_dif_5_GDPgrowth lag_dif_5_elc lag_dif_5_vae, re vce(robust)

mi estimate: xtreg logmortmale lag_dif_5_mpa lag_dif_5_GDPgrowth lag_dif_5_elc lag_dif_5_vae, re vce(robust)

mi estimate: xtreg logmortfemale lag_dif_5_mpa c_year lag_dif_5_GDPgrowth lag_dif_5_elc lag_dif_5_vae, re vce(robust)

mi estimate: xtreg logmortmale lag_dif_5_mpa c_year lag_dif_5_GDPgrowth lag_dif_5_elc lag_dif_5_vae, re vce(robust)

*Sensitivity Analysis: Differencing Estimator

mi estimate: regress lagdifmale lag_dif_5_mpa clagyear lag_dif_5_GDPgrowth lag_dif_5_elc lag_dif_5_vae, vce(robust)

mi estimate: regress lagdiffemale lag_dif_5_mpa clagyear lag_dif_5_GDPgrowth lag_dif_5_elc lag_dif_5_vae, vce(robust)
